# GEROFIT TO HOME: REDUCING BARRIERS TO GROUP EXERCISE TO RURAL AND NONRURAL OLDER ADULTS IN A SUCCESSFUL FASHION

**DOI:** 10.1093/geroni/igac059.1273

**Published:** 2022-12-20

**Authors:** Neil Gregor, Eva-Marie Christman, David Crivello, Jamie Schukle

**Affiliations:** Veteran Affairs, Martinez, California, United States; Veteran Affairs, Redding, California, United States; Veteran Affairs, Martinez, California, United States; Veteran Affairs, Martinez, California, United States

## Abstract

Transportation, scheduling and travel distance can present as barriers to participation in facility based exercise programs. Gerofit to Home(GTH) eliminates aforementioned burdens commonly associated with in-person programs. Understanding participant satisfaction and retention in rural(R) and non-rural(NR) groups assists in planning to better serve these populations with group exercise. This study aims to explore GTH participant satisfaction and retention in over 100 participants, through 3 months, for veterans over 65yo in rural and non-rural areas. Current results illustrate “Highly Satisfied” in majority of both R and NR groups. Retention at 3 months, in both groups, and factors associated with 3-month retention will be discussed in this presentation. Further research into the reasoning for participant stoppage prior to 3 months will aide in understanding how to better cater group exercise to these groups. Satisfaction scores in this study endorse further efforts for virtual group exercise in older adults.

